# Novel Size-Tunable and Straightforward Ultra-Small Nanoparticle Synthesis in a Varying Concentration Range of Glycerol as a Green Reducing Solvent

**DOI:** 10.1021/acsomega.3c02697

**Published:** 2023-07-26

**Authors:** Iqra Munir, Gurkan Yesiloz

**Affiliations:** † National Nanotechnology Research Center (UNAM), Bilkent University, 06800 Cankaya-Ankara, Türkiye; ‡ Institute of Material Science and Nanotechnology, Bilkent University, 06800 Cankaya-Ankara, Türkiye

## Abstract

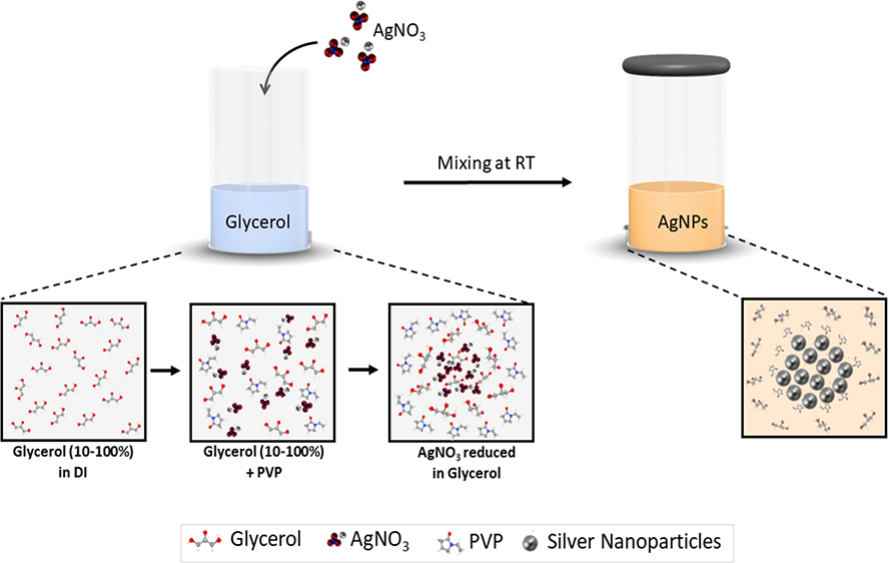

Despite all the possibilities available so far for the synthesis of nanoparticles (NPs), synthesizing ultra-small (<10 nm) monodispersed particles is still demanding. Getting a particular size with a straightforward method is a trial-and-error approach. To explore this prospective, in the current study, we have introduced a protocol which offers a varying concentration range of glycerol to successfully generate the NPs of repeatable and consistent particle size in each synthesis, thus giving an alternative from lengthy tentative preparations and/or testing protocols. Since synthesizing controlled sized nanoparticles in aqueous medium is somewhat difficult as the balance of particle growth and nucleation is challenging to control, herein, we used a polyol method with glycerol both as a solvent medium as well as reducing species for silver nitrate, as an example model ion source, to execute the nanoparticle synthesis. In order to maintain the stability of the synthesized NPs, polyvinylpyrolidone (PVP) was added as a stabilizer. The synthesis, monodispersity, and stability were confirmed using techniques such as UV–vis spectroscopy, Fourier transform infrared spectroscopy (FTIR), dynamic light scattering (DLS), and X-ray powder diffraction (XRD), while morphological analysis and ultra-small size validation were conducted using TEM, SEM, and AFM. Interestingly, in the various concentrations of glycerol solution used (10–100%), we have observed a tunable linear size range to obtain ultra-small nanoparticles (<10 nm) up to 60% glycerol, while further increasing the glycerol component increased the size approximately to ∼160 nm, providing tunable properties in this synthesis procedure. Hence, this study provides a distinct possibility to obtain ultra-small nanoparticles with a tunable size feature for further applications in numerous fields.

## Introduction

Regardless of the constant progression in the metal nanoparticle (NP) synthesis, uniform distribution of particle size and controlled size tunability in a designed single protocol yet remain the critical features. For instance, in the synthesis of nanoparticles, the size and shape of NPs are among the key components^1^ for NP-based drug delivery, since there is a high probability of smaller sized particles to efficiently escape the body’s natural clearance system, with an extended circulation time in the blood.^2,3^ However, when it comes to NP synthesis approach, the existing literature needs to be improved further to highlight simple single-step protocols that can generate nanoparticles of ultra-small size with high reproducibility.

On the other hand, in the search of environment-friendly solvents and processes for the synthesis of nanoparticles, glycerol has been notified as a “green chemistry solvent” because of its nontoxicity, biodegradability, and renewable characteristic, as well as its ability to dissolve a variety of compounds (having poor water solubility) with ease, including organic compounds, inorganic salts, and acids/bases.^4^ Synthesis of metal nanoparticles in glycerol has also gained attention, with reaction preferences in alkaline medium and at high temperatures, attributed to the high-temperature resistance of glycerol,^5,6^ thus revealing it as a solvent medium as well as a reducing compound for NP synthesis.

Among all the well-recognized NP synthesis methods, metal reduction is preferred by either NaBH_4_, hydroxylamine hydrochloride,^7^ or citrate, with alternative choices of ascorbic acid,^8^ dextrose,^9^ and/or NaOH.^10^ Out of all these approaches, a major drawback is that none of these are yet capable of producing nanoparticles of high monodispersity with respect to the shape and size of NPs in one simple step. Additional steps like centrifugation, sonication, heating, and pH and/or temperature conditions in the reaction^11^ are key to reach the desired size during the synthesis. Other than that, for the purification of such colloidal heterogeneous mixtures, separation techniques like density gradient centrifugation can be used thereby for the selection of subpopulations having preferred properties. However, these approaches revolve around the real monodispersity issue, but unfortunately none of it completely fulfills this demand.^12^

Theoretically, to attain particle size with monodispersity, one of the crucial prerequisites is a quick homogeneous nucleation, and the selective heating profile has a direct impact on growth and nucleation time in NP synthesis, which results in the homogeneity of the synthesized particles.^13^ Interestingly, following a simple protocol like the current study, both homogeneity as well as ultra-small particle-sized NPs can be obtained at ambient temperature, which could have more than one reason: for example, the impact of solvent (glycerol) and the presence of stabilizing agent (PVP),^14^ thus modifying and controlling the nucleation profile to achieve homogeneous particles at variable concentrations of the solvent.

Although metal nanoparticles have been synthesized in glycerol within limited experimental conditions, including high temperatures, alkaline pH conditions, and the irradiance of ultraviolet light, herein, we report that the metal nanoparticles have been formed in glycerol under completely green conditions, for example, room temperature, neutral pH conditions, and without ultraviolet irradiation. It is attributed that aldehydes and free radicals are generated in glycerol via oxidation, which operates as reducing species, and in such reactions, the oxidation can be controlled using specific additives or stabilizers, e.g., PVP, which is known to have a critical role in controlling the oxidation of glycerol in many ways, for instance, stabilization of NPs, control over reaction kinetics, particle size, morphology control, and so forth. By stabilizing the NPs, it helps control the oxidation of glycerol by ensuring a stable environment for the reaction. Also, in the synthesis conditions, by reacting with oxygen or other oxidizing species present in the reaction environment, it can reduce their concentration and minimize their impact on the oxidation of glycerol. Even though, in the present circumstances, there are less reports in describing the actual glycerol reduction mechanism for metal ions, with a specific temperature requirement at high degrees, evidences in the literature also correlate this reduction in the presence of an aldehyde group, i.e., glyceraldehyde generated from glycerol (alcohols) at these temperatures.^15^ Nevertheless, in recent findings, the nondependency of high temperature for the metal reduction in glycerol has also been confirmed with the production of metal NPs at ambient temperature.^4^

Remarkably, in biomedical or clinical applications, some metal NPs (e.g., gold, silver, and iron), when prepared using biosynthesis (green synthesis) protocols and neutral pH conditions, can be more suitable, attributed to the demand of developing environmental friendly procedures for nanoparticle synthesis.^16^ Knowing the commercial applications of nanoparticles in medical and pharmaceutical sciences, these NPs when synthesized naturally become more suitable for medical applications by reason of greater biocompatibility than the ones synthesized chemically.^17^ Moreover, synthesizing monodispersed NPs is also critical to ensure reliability when used in biological experiments and to permit a smooth translation and/or utilization into the clinics.^18^ However, all these applications directly depend on the properties of synthesized NPs, specifically the size of particle, charge on the surface, and size distribution (monodisperse/polydisperse).^19^

In the current study, silver, as a model metal source, was selected due to many reasons. For instance, generally, it is more challenging to synthesize silver NPs of high monodispersity with simple procedures than preparing NPs of similar features from other metals.^20^ Besides, nanoparticles of noble metals, in particular, silver, have gained massive attention in the scientific world, owing to their physical and chemical properties that vary considerably from the similar substances when in bulk. Silver NPs have not only been investigated in engineering experimental domains, for example, catalysis,^21−23^ fabrication of high-conductivity elements in printed electronics,^24^ construction of detectors due to the existence of surface plasmon,^25−27^ surface-enhanced Raman spectroscopy substrates,^28^ and so forth, but also investigated in medical world due to their surprising activities as antibacterial agents.^29^

In our study, we have utilized an altered mechanism for silver NP synthesis, for which silver nitrate was used as a metal ion source, utilizing polyol (glycerol) as a solvent medium with varying concentrations (10–100%) at room temperature (RT). Additionally, polyvinylpyrrolidone (PVP) was added as a stabilizing/capping agent as well as a size controller to obtain ultra-small silver NPs. With our protocol, we have acquired a varying concentration range of glycerol (10–60%), where obtaining NPs of ultra-small size in a single step has become possible, with great reproducibility and size tunability. To our best knowledge, this study reports for the first time the synthesis protocol which offers a range of glycerol as a solvent medium, where changing the concentration of glycerol carries the opportunity to produce silver NPs of desired small size and tunability, without additional lengthy steps of synthesis protocols. For this purpose, the synthesis and characterization of NPs were confirmed using various techniques such as UV–vis spectroscopy, FTIR analysis, DLS, and XRD, while size and morphological analyses were conducted using TEM, SEM, and AFM, thus confirming the synthesis of silver NPs with an offered range up to 60% glycerol, to obtain <10 nm particles in a single step at room temperature. Hence, with the mentioned protocol in this study, the production of ultra-small-sized nanoparticles at a large scale, for commercial or industrial applications potentially, is possible without additional time-consuming and costly processes (such as the need of high temperature, pH adjustment, and/or sonication).

## Experimental Section

### Chemicals and Apparatus

Silver nitrate (AgNO_3_, Merck), glycerol (C_3_H_8_O_3_, ≥99%, Birpa), and polyvinylpyrrolidone (PVP) (average *M*_W_ 55,000, Sigma Aldrich) were used as it is without additional purification, and deionized water (DI, 18.2 MΩ cm/MilliPore) was used as the aqueous solution source to synthesize silver nanoparticles. All glassware and magnetic beads were treated with “aqua regia” solution (acidic mixture of concentrated hydrochloric acid (HCl––Merck) and nitric acid (HNO_3_––Sigma Aldrich) at a 3:1 ratio (v/v) for at least 15 min and air-dried before and after the synthesis of silver NPs.

### Silver Nanoparticle Synthesis in Glycerol

**Scheme 1:**
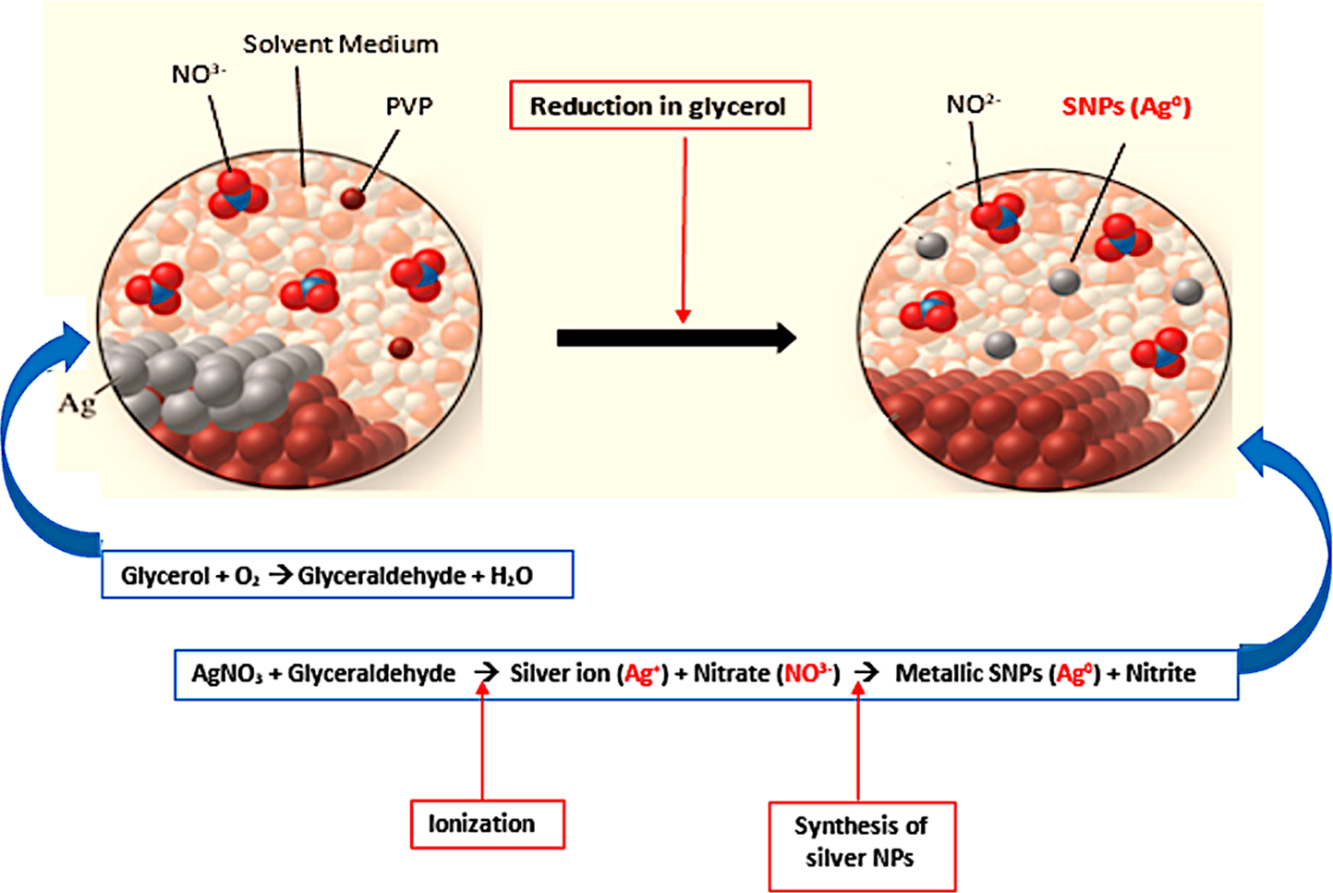
Silver NP (SNPs) Synthesis with Silver Nitrate in Polyol (Glycerol) Medium and PVP under Neutral Conditions

For the synthesis of silver NPs, a wide range of glycerol solutions were prepared (10–100%) by mixing the calculated volume of glycerol with water for individual percentage (Scheme 1). The solution was kept for thorough mixing using magnetic beads until both solutions were homogeneous. Further, to this mixture, 0.4% PVP (0.02 g) was added and left until the powder was completely dissolved. Since PVP has high solubility in water, the solutions with increasing concentrations of glycerol took comparatively more time (∼3 h) to get a transparent homogeneous solution of PVP in glycerol (e.g., 15–60 min for 10–50% glycerol solutions and 2–3 h for 60–100% glycerol solutions w/v) at room temperature.

The next step was the addition of 1.65% silver nitrate (∼0.085 g) to all solutions prepared previously. The final mixture was left for mixing (∼3 h) using magnetic beads at room temperature (RT) (20–25 °C) until the transparency of the solution started turning into a noticeable (light yellow) color, confirming the synthesis of silver NPs. Later, the magnetic beads were taken out, and solutions were left on the laboratory bench (without mixing) overnight to observe the stability of the synthesis reaction. Interestingly, all solutions had a color appearance from light yellow to brown with respect to the increasing concentration of glycerol as a solvent medium. The prepared solutions were further used for characterization techniques.

### Characterization of Silver Nanoparticles

#### UV–Vis Instrumentation and Methodology

The synthesis of silver NPs was observed physically by color change and further confirmed using a UV–vis spectrophotometer (Cary 100 Bio) in the wavelength range from 200 to 700 nm against equimolar glycerol and PVP solution as blank, as well as reference solvent. For the analysis, quartz UV cuvettes with a path length of 10 mm were used, and the volume requirement was 3.5 mL for each sample and reference solvent. The spectrometer uses a double-beam configuration equipped with a 1 + 1 cell changer. Samples were used as such without any further dilution.

#### Dynamic Light Scattering Instrumentation and Methodology

The size measurement, monodispersity, and potential of NPs were observed using a DLS instrument (Malvern Nanoseries-ZS). Measurement was conducted using disposable micro- cuvettes, cleaned with DI water and air-dried before use. All the measurements were performed at defined parameters by adding solvents’ viscosity and refractive index information at room temperature. To obtain the average hydrodynamic diameter (Z.avg) and polydispersity index (PDI), cumulative analysis algorithm was applied. The PDI indicates the width of the size dispersion, and usually a high PDI value specifies the aggregation status in the particles that results in size heterogeneity.^30,31^

#### Fourier Transform Infrared Spectroscopy

FTIR spectroscopic (Vertex 70) measurements were conducted from 400 to 4000 cm^–1^ in attenuated total reflection (ATR) mode on a diamond disc, with 32 accumulations, to observe the functional group variation in glycerol before and after silver NP synthesis and to investigate the coordination of silver NPs with PVP. Sample analysis was conducted by FTIR spectroscopy by dropping a 5 μL sample on the diamond disc. Measurement was conducted by recording the background spectra as blank, to subtract it from sample measurements.

#### TEM Instrumentation and Methodology

Further confirmation of NP size distribution was attained by diameter measurement using electron micrographs from a transmission electron microscope (TEM, Tecnai G2, FEI). The TEM samples were made ready by drop-casting 2 μL of individual silver NPs in glycerol solution onto a Formvar–carbon-coated 400 mesh copper TEM grid (Agar Scientific, UK) at RT until being dried and imaged on a FEI Tecnai TF20 FEG high-resolution TEM system, operating at 200 kV. For the calibration of magnification as well as the scale bar for TEM images, a standard cross-grating was used. For the calculation of the average size and standard deviation of silver NPs, an image-processing tool (Image J 1.50i) was used, with around 50 particles in the TEM images.

Scanning-TEM-HAADF (high-angle annular dark-field) images and STEM-energy-dispersive X-ray spectroscopy (STEM-EDS) spectrum images were obtained with a Nion UltraSTEM 200-X instrument. The magnification calibrations for the HAADF images were checked against the gold nanoparticle lattice spacing. The EDS spectrum images were measured using EDX AMETEK, with an active area of 30 mm, and analyzed with the TIA (TEM Imaging and Analysis) software. The STEM-EDS maps are drawn with raw counts, using the Cu peaks.

#### SEM Instrumentation and Methodology

Due to the specificity of TEM for the observation of very low particle sizes (<10 nm), for the morphological analysis of particles above 100 nm, SEM images were obtained by a FEI Quanta 200F system, which is a field emission gun (FEG-SEM) operated in environmental (ESEM) mode, at 20.0 kV accelerating voltage. Samples were washed with ethanol, dropped on glass slides, and air-dried before taking images. The average size was measured from ∼200 particles (more than that measured by TEM due to its low scale bar) of as-obtained silver NPs to provide statistical significance.

#### XRD Measurements

In order to perform XRD analysis, identical samples were used and measured by an XRD (Malvern Panalytical’s X’Pert PRO) system. The spectra of powder XRD were acquired using a ceramic X-ray tube with Cu Κα (λ = 1.54178 Å) anode at 45 kV and 40 mA, with a reflection Bragg–Brentano geometry. Samples were prepared by drop-casting silver NPs on a nonbackground silica surface, followed by air-drying overnight. The samples were scanned over a 2θ range of 20–90° with a step size of 0.0262°, and a high-sensitivity PIXcel 1D detector was used to collect the high signal-to-noise spectra. The collected XRD data were background-subtracted using the X’pert data collector software package.

#### AFM Measurements

An atomic force microscope (Park System XE-100E) was used to measure the surface morphology of nanoparticles. AFM images were collected using aluminum coating cantilevers (PPP-NCLR) with a force constant of 48 N/m and a standard tip shape (radius: <10 nm) in noncontact mode. To arrange the slides for AFM sampling, briefly, the glass slides were cleaned with ethanol, and then water, and were air-dried carefully. Next, 5–10 μL from the silver NP sample was dropped on a glass surface and dried in the clean environment. The ready samples were observed under AFM to record the size, morphology, and average surface roughness (Ra) of the synthesized silver NPs in 100% glycerol.

## Results and Discussion

### Synthesis of Ultra-Small Silver Nanoparticles in Glycerol

**Figure 1:**
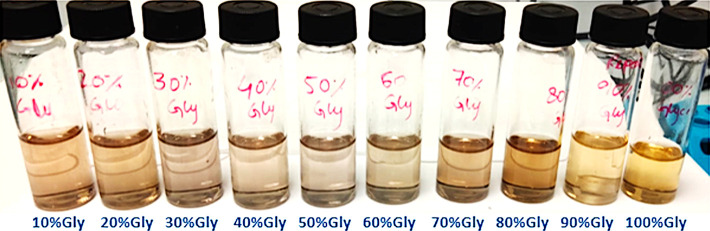
Silver nanoparticle synthesis in varying concentrations of glycerol (10–100%).

One-step synthesis of silver NPs in varying concentrations of glycerol (10–100%) as a reducing agent was conducted at RT (Figure 1). During the synthesis step, a sustained reaction kinetics started upon mixing silver nitrate (metal source) with glycerol (as a reductant)–PVP solution, resulting in the direct reduction of Ag^+^ to produce metallic Ag atoms.

As the process initiated, the freshly reduced Ag (silver) atoms served as the nuclei of the NPs, which kept on growing continuously as the processing time exceeded, catalyzing the reduction of the remaining Ag metal ions present in the solution.^32^ Typically, such coalescence of atoms results in the metal cluster formation, which needs stabilization by specific surfactants, polymers, or ligands (for instance, PVP in the current study). The synthesis continued during the course of the investigated period, as shown by the appearance and the progressive enhancement in the color intensity at each percentage. The color thus indicates the formation of NPs in a solution.^32^

The distinctive colors of metal NPs are principally due to the plasmon absorption phenomenon. It appears when a conduction in electrons is observed due to the incident light on the NP surface, resulting in the absorption of electromagnetic radiation.^4^ Such spectrum of varying colors has been observed during silver NP synthesis in glycerol (Figure 1). A prominent increase in color intensity was noticed with regard to the increased glycerol content of the solution, thus confirming the variation in color due to the difference of reduction potential provided by glycerol in each composition,^4^ thereby affecting the size as well as absorption capacity at each percentage (i.e., 10–100%). Another correlation of the metal NP color has been defined with respect to shape/size of the synthesized particles along with the refractive index (RI) of the surrounding solvent medium.^33^ Since this change in the color of the solution is slower in comparison to the classical Lee and Meisel^34^ method, it leads to a hypothesis that glycerol drops the reduction/nucleation speed and hence highlights the significant role of the solvent in the whole reaction process.

From the photographs of silver NPs obtained at increasing glycerol concentrations in Figure 1, it can be visibly observed that the color appearance of the silver NPs changes from light yellow to sharp yellow. However, the presence of PVP resulted in increased stability and/or reduced dispersity of the obtained NPs in the solution, which is confirmed by the presence of highly specific sized particles in a linear range at each percentage. This change in color also relates with the increase in particle size at higher percentages (i.e., 60–100%), which is also evidenced and complemented by the data of UV–vis spectroscopy, DLS, and TEM images.

Theoretically, out of the three mechanisms defined with reasonable explanations for metal ion reduction in glycerol, namely reduction by aldehydes, catalytic reduction, or reduction by alkoxides, the former two have reaction conditions of high/room temperature, respectively, and/or an alkaline medium.^35^ It has been noted that silver ions in alkaline conditions can easily react with OH (hydroxide ions) to generate particles of Ag_2_O (silver oxide), while aldehyde formation is generally linked with the dehydration of glycerol in alkaline conditions when heated at high temperatures.^36^ On the other hand, in alkoxide reduction (formed from alcohols, ketones, or aldehydes by hydroxide ions), there has not been a necessity to convert glycerol to aldehydes, since the metal reduction can be proceeded directly by alkoxides generated from these alcohols.^37^ Hence, alkaline conditions and high temperature are no more the compulsion for glycerol to be used as a medium, as evidenced from the current experimental results and the recently discovered strategy by Liu et al., where synthesis has occurred in neutral conditions and at room temperature, making it fairly a new mechanism in the synthesis of nanoparticles without an additional strong reducing agent.^4^

### UV–Visible Absorption Spectra of Ultra-Small Silver Nanoparticles

**Figure 2:**
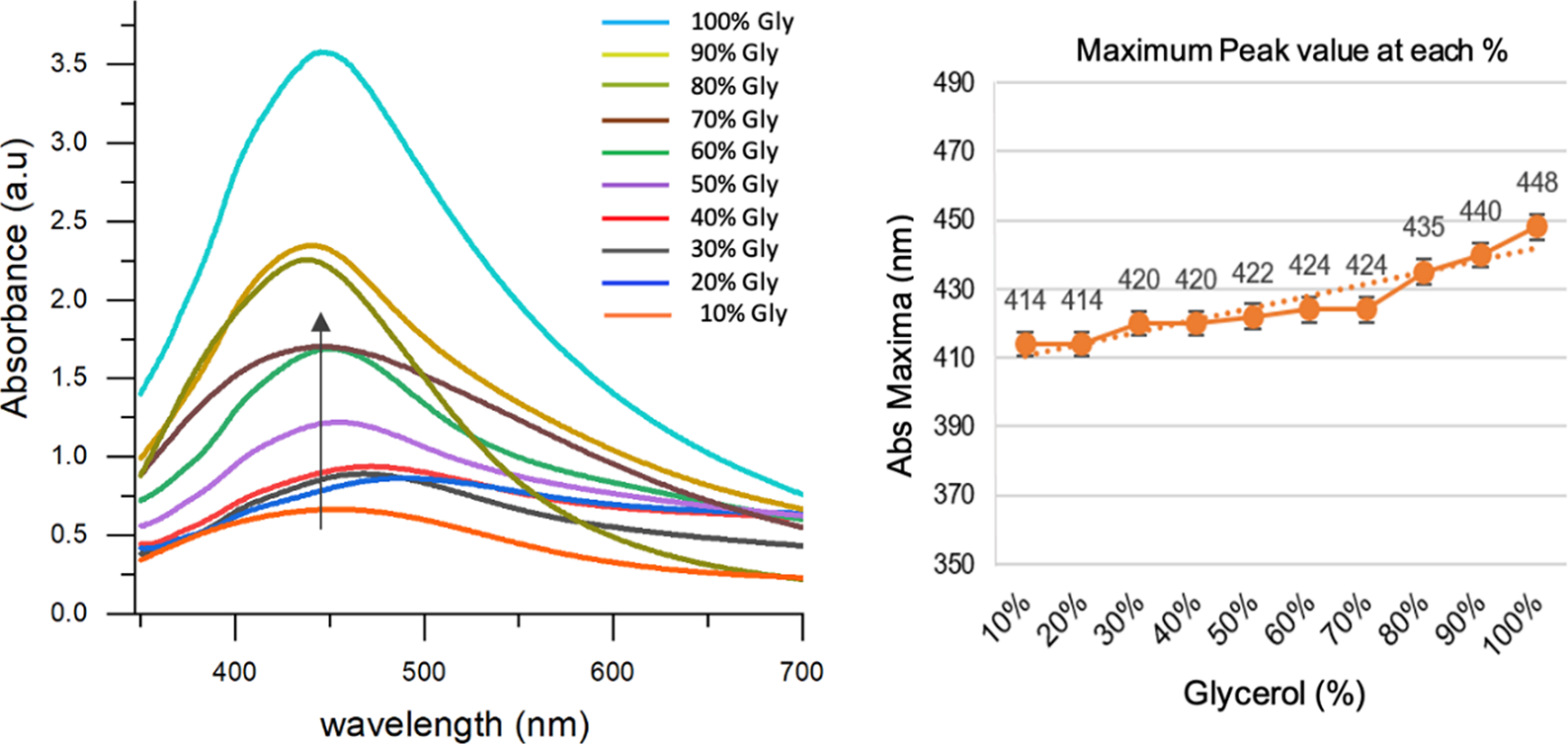
UV–vis absorption spectrum of synthesized silver NPs (left) and graphical illustration at the absorption maxima to observe linearity (right) in varying concentrations of glycerol (10–100%).

The study of the effect of glycerol as a reducing agent on the synthesis of silver NPs was further followed by UV–vis spectroscopy using absorbance measurements in the presence of PVP. Figure 2 illustrates the UV–vis spectra of the synthesized particles, and peak maxima at each concentration are stated in Table 1. The spectrum indicates the appearance of optical absorbance positions between 410 and 450 nm, which correlates with the surface plasmon of silver NPs.^38^ Here, the peak positions of each concentration of glycerol (10–100%) undergoes a sharp blue shift at a low glycerol concentration (i.e., 10–30%), with a trivial switch to red shift at increased glycerol proportions (i.e., from 50 to 100%), consequently giving a static peak for the middle range of glycerol (50–70%), while a significant red shift at a high glycerol range (i.e., 80–100%) (Figure 2). The results are in agreement with the theoretical data that a blue shift in the UV spectrum is related to the small size particles, while bigger NPs will have a peak with the red shift.^39^ However, the particular position of absorption due to plasmon may also rely on multiple factors like size and shape of the particle, type of solvent, as well as the capping agent.^39^ However, monitoring the plasmon peak as a function of time can provide valuable information about the reaction rate in nanoparticle synthesis, but that would be more appropriate when done along with factors that may influence the reaction rate, e.g., temperature, concentration, and/or any catalysts or additives for each percentage of glycerol (i.e., 10–100%), at regular intervals, to monitor the plasmon peak position and intensity over time. In the current study, since most of the parameters in all samples were kept the same (i.e., concentrations of glycerol and PVP, room temperature), with only the glycerol percentage (10–100%) as a variable, the reaction rate has not been calculated through the plasmon peak as a function of time, and the data have been presented as UV spectrum (indicating red shift at varying percentages) along with a separate graph showing peak maxima at each percentage. However, with the intention to highlight the size specificity in each composition (10–100%) of glycerol, a linearity curve was generated (Figure 2) using the absorbance values of silver NPs at wavelength maxima to find out the range where size variation appears to be the least altered. To our attention, a consistent absorption pattern was observed when up to 60% glycerol was used as a solvent medium, thus providing a concentration range of glycerol in the synthesis of NPs, while above 70%, the absorption started increasing, indicating the improved absorption relevant to the increased size of NPs. The results herein endorse the protocol offering a linearity range to successfully generate ultra-small nanoparticles (<10 nm) in a single step. Furthermore, the size of the silver NPs synthesized at various glycerol compositions was acquired using DLS and TEM measurements.

**Table 1:** Particle Size Distribution as a Function of Glycerol Percentage

samples	glycerol (%)	PVP concentration (M)	particle size (nm)	PDI (%)	λ_max_ (nm)
1	10	0.003	1.70	0.323	414
2	20	0.003	2.45	0.442	414
3	30	0.003	3.68	0.588	420
4	40	0.003	5.27	0.341	420
5	50	0.003	6.97	0.519	422
6	60	0.003	7.32	0.493	424
7	70	0.003	10.09	0.315	424
8	80	0.003	15.87	0.298	435
9	90	0.003	67.95	0.192	440
10	100	0.003	159.6	0.397	448

### DLS of Ultra-Small Silver Nanoparticles

**Figure 3:**
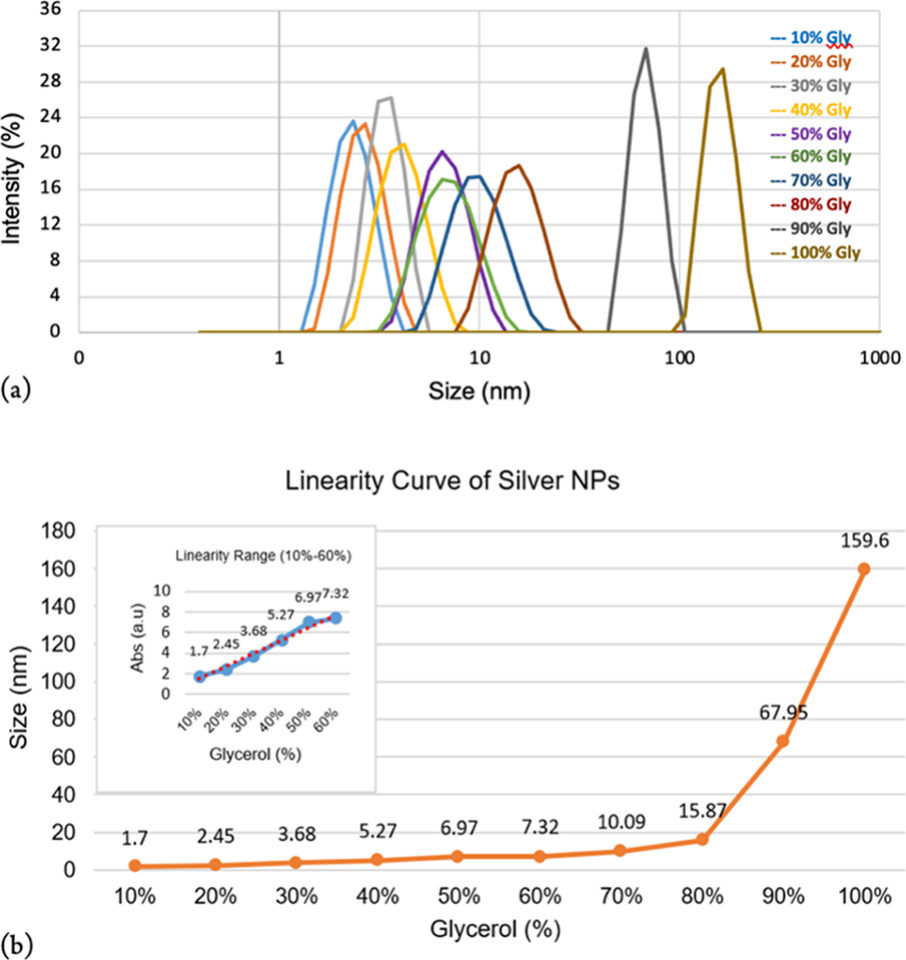
Size distribution analysis of silver NPs (10–100%) with DLS and (a) linear curve obtained from particle size data (b).

A DLS measuring tool to calculate the hydrodynamic size (Rh) of NPs was used to evaluate silver NPs in glycerol (10–100%) through the polydispersity index (PDI) and average diameter (nm). We performed a comprehensive analysis of the silver NPs synthesized at ambient temperature. Figure 3a demonstrates an overlay plot of the hydrodynamic diameter in all ten compositions (10–100% glycerol) of silver NPs. Interestingly, among NPs, a highly stable control of particle size was detected up to 60% glycerol. Here, a significant proportion of NPs presented particle diameter of <10 nm with a PDI range of 0.3–0.6%.

However, the size started increasing from 10 nm up to 160 nm with a comparatively low PDI range of 0.1–0.3%, at higher glycerol concentrations (>80–100%) (Table 1). This variation in particle size could probably be accredited to the varying concentration of the precursor (for instance, glycerol) in the confined reaction, making sense that increasing the concentration of glycerol consequently increased the availability of the reducing agent, hence reducing the silver nitrate to its fullest at high concentrations. This eventually effected the size distribution of particles from low to high.

To demonstrate the linearity range observed in this study, a linearity graph was also plotted from the particle size acquired from DLS at each percentage. As shown in Figure 3b, the size remains <10 nm up to 60% glycerol, with a stable peak in DLS (Figure 3a), and starts increasing at a percentage of 70% and above of glycerol. Since DLS is an extremely sensitive technique to determine the existence of agglomerates, all the experiments were performed in triplicates in order to remove any uncertainty of the measurements with very low particle size (<10 nm). Thus, this protocol is one of a kind to offer small-sized particle synthesis over a range of solvent percentages, with high reproducibility.

Previously, Tobler et al. attempted to observe the growth and nucleation of other metal NPs using DLS and SAXS measurements, considering the efficiency of DLS to display the initial step of the aggregation progression in their particles.^40^ However, the data were further verified through TEM/SEM regarding the particle size, even though these microscopic techniques under some conditions (like the sample exposure to high vacuum or dehydration of the sample) may yield smaller sized NPs as compared to DLS/SAXS.

**Figure 4:**
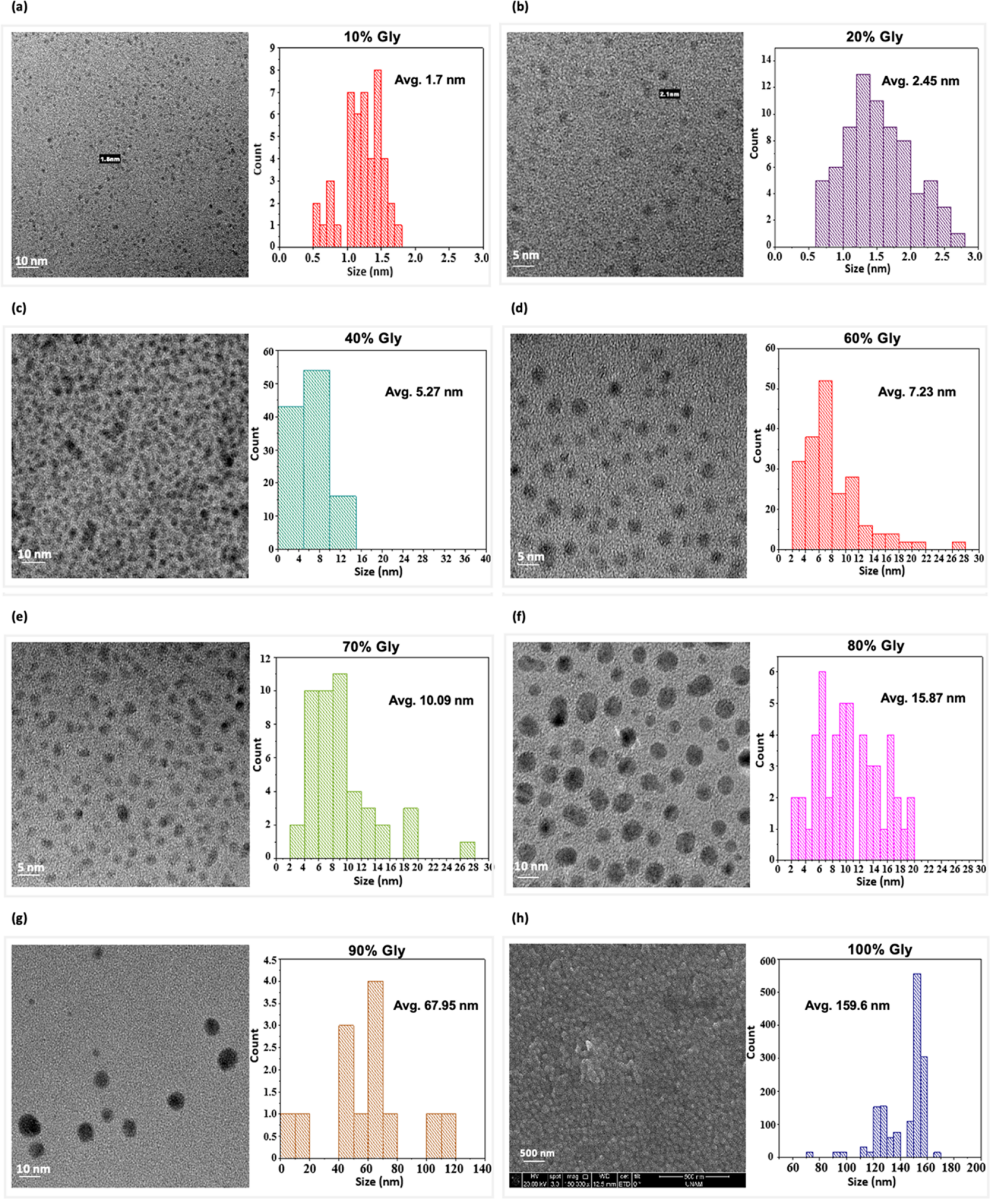
TEM/SEM micrograph images of silver NPs in glycerol (10–100%) and the corresponding size measurements. (a) 10% glycerol (Avg. 1.5 nm); (b) 20% glycerol (Avg. 37.22 nm); (c) 40% glycerol (Avg. 37.24 nm); (d) 60% glycerol (Avg. 36.44 nm); (e) 70% glycerol (Avg. 36.32 nm); (f) 80% glycerol (Avg. 37.24 nm); (g) 90% glycerol (Avg. 36.44 nm); and (h) 100% glycerol (Avg. 159.6 nm).

Relating to the size measurement, it is noted that TEM solely provides the detailed structural information for samples in a dry state; therefore, a slight difference may occur from the similar sample when in liquid state. In contrast, DLS denotes the dispersed sample state, with the necessity of correct refractive index specifications of the solvent, to acquire reliable nanoparticle measurements (Figure S1). For these reasons, it is worthwhile to include both as complementary techniques when evaluating the particle size.^41^ Our presented DLS data were in agreement with the images of silver NPs obtained from TEM (Figures 4 and S1, S2).

### TEM and SEM Photographs of Ultra-Small Silver Nanoparticles

In order to complement the size observed on DLS, the samples were subject to TEM analysis to authenticate the obtained data for the current synthesis. Figure 4 shows the TEM images of silver NPs synthesized in glycerol (10–100%) + PVP, which predominately show small-sized particles having spherical morphologies, with an overall size range of 2–7 nm for <60% glycerol preparations while 10–160 nm for glycerol solutions of 70% and above. Using STEM-HAADF- and EDS-derived elemental mapping images, we validated that the presence of silver NPs maintained a general spherical shape and well-dispersed appearance (Figures 4 and S3). In our analysis, TEM images of silver NPs in increasing concentrations of glycerol (10–100%) exposed the NPs (*Z* = ∼50) to appear as black figures, on a gray cluster background from the carbon support. Among all the percentages, about 70% of the silver NPs hold a size of lower than 10 nm.

For the particle size distribution, subsequent histograms were acquired using Image J software. By denoting the results in Figure 4, an increasing trend has been observed for average silver NP size from low (10%) to high (100%) glycerol concentrations. Moreover, along with the higher percentage of glycerol, the histograms of particle size also show broadening of the particles through the overall wider size distribution from 0.5–20 to 0.5–160 nm, and particles as small as 1.8 nm and as large as 159 nm were being found in the samples synthesized at the lowest-to-highest glycerol concentrations. Change in shape from spherical to quasi-spherical was also observed, especially at higher concentrations (80–100%) of glycerol. Since, with the increasing glycerol content, increased availability of the reducing agent was noticed, it is believed that the remaining Ag^+^ ions at higher values continued to grow along with the existing silver NPs, thus altering the morphology of the particles in later stages. The results somehow correlate with the phenomenon of law of mass action where the reaction rate is directly proportional to the reactant concentration.^42^

Moreover, in a study by Steinigeweg et al., they used a mixture of glycerol–water: citrate (40%:3%) as a viscous component at 95 °C alone and after the addition of other reducing agents (e.g., ascorbic acid, NaBH_4_, and hydroxylamine hydrochloride) to compare it with the classical method of Lee and Meisel^34^ where AgNP synthesis occurs in water. Their results also indicated small, monodispersed particles in glycerol, with a size reduction from 83.9 to 30 nm in their proposed composition from the former method. However, addition of any external reducing agent and/or hydroxyl group molecules (ethylene glycol, agarose, or sucrose) brought aggregation and polydispersity in the silver colloid particles.^12^ Unfortunately, their study limited the synthesis of silver NPs up to 20–30 nm with the given protocol, which reached a higher size (40–110 nm) upon the addition of ascorbic acid and PVP, thus making our approach more effective in obtaining silver NPs of tunable size range in a one-step synthesis protocol. Another study by Jacob et al.^43^ also portrayed the polyol method (with ethylene glycol) for the synthesis of silver nanoparticles, through varying the compositions of ethylene glycol:water, and obtained a size up to 20 nm as the lowest value.^43^ Our results also correlate with the previous findings by Agnihotri et al., where they used a different reduction strategy to obtain a range of nanoparticles (5–100 nm) with high reproducibility, but their method was composed of chemical synthesis at variant temperatures,^44^ thus empowering our results to obtain an even smaller size range (i.e., as low as <2 nm) with high reproducibility, and provided a linearity range of synthesis even at room temperature.

Additionally, in our synthesis with 100% glycerol, the size that appeared in DLS was 159 nm (Figure 3), which can preferentially be visualized under SEM. Thus, SEM analysis was conducted at this percentage to monitor the size as well as appearance of silver NPs (Figure 4h). To our interest, the results correlated well with the DLS observations, giving a wider distribution of size from 70 to 160 nm in Image J software histogram, having much of the particles lying in the desired size range (Figure 4h). Our results look alike with those of Devaraj et al., who prepared AgNPs with a green synthesis method and observed uniform particles of around ∼61 nm under SEM.^45^ Similarly, Sinha et al. attempted the preparation of fine silver powder through the glycerol process, with high purity and uniformity in particle morphology at high temperatures. Their results showed the synthesis of spheroidal silver powder, with the average particle size of 1.5–11 μm, having a narrow size distribution.^46^ Unlike their images, our results show much stabile particles with even distribution throughout the plane, emphasizing the effect of PVP as a strong stabilizing agent.

Therefore, it can be interpreted that with the increasing glycerol percentage, there should be an increase in the reaction rate as well, and with such increment in the rate, the reduction and/or consumption of silver ions should also speed up.^42^ However, our results point toward the fact that although there is a higher amount of glycerol in the medium (i.e., more reducing agent available to conduct the particle synthesis), due to the high viscosity of the surrounding medium, the reaction rate decreases at a high glycerol concentration, resulting in sequential big-sized particle synthesis (i.e., from 2 to 160 nm) with respect to the glycerol percentage (i.e., 10–100%) in the medium. Our results are in agreement with the theoretical justification that during the process of synthesis, too fast rate of reaction allows the quick formation of more metal nuclei and hence the small particles are obtained. On the contrary, a very slow reaction rate can result in particle agglomeration.^47,48^ In order to prevent any such agglomeration or aggregation, PVP was added in our synthesis batch to stabilize the synthesized particles at all percentages.

Thus, among many other limitations during the synthesis of NPs, appropriate reducing agent selection is one of the key factors as it has a direct dependency on the shape, size, as well as particle size distribution obtained at the end of the reaction.^48^ Moreover, since the characterization of nanostructures has always been an important yet difficult-to-handle matter, TEM has proven and supported to be an important technique to make available the reliable data for most of the nanomaterials at a very low range (i.e., up to 1 nm), thus also giving the possibility to overcome the SEM magnification limitation range in nanoparticle analysis. Likewise, with the lens aberration correction feature in the TEM system, direct imaging of the sample’s atomic structure with the point resolution of as low as 0.24 nm or better can also be obtained.^49,50^

### Infrared Spectra of Ultra-Small Silver Nanoparticles

**Figure 5:**
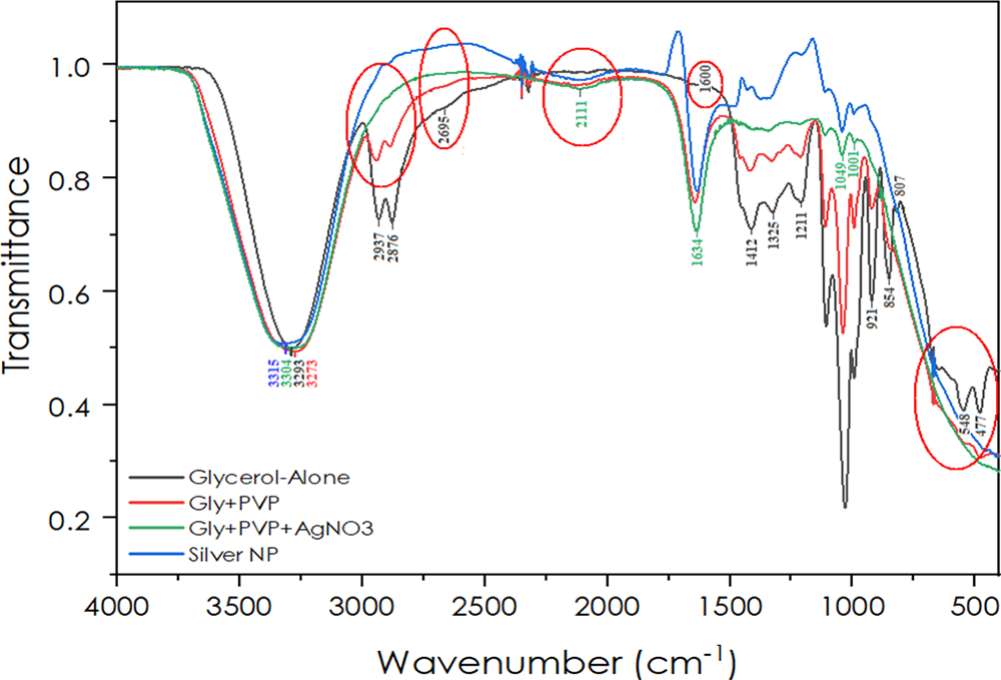
FTIR absorption spectrum of silver NPs in glycerol.

In the current study, FTIR was measured to categorize the potential molecules in glycerol responsible for reducing silver nitrate in order to synthesize silver nanoparticles. The FTIR spectrum not only provides sufficient information about the functional groups of the compound as well as their interactions with metals (e.g., silver) but also gives the idea about the molecular surroundings of the organic substances around the NP surface.^38^Figure 5 displays the FTIR absorption band patterns of silver NPs synthesized in glycerol (10–100%) with PVP. A comparative analysis of glycerol and PVP (alone and in conjugation) was also done (Figure S4) to observe the role of these compounds in varying the profile of silver NPs during the synthesis and to identify the potential molecules accountable for the efficient stabilization and capping of the metal NPs synthesized with glycerol as a reduction medium. The results indicate the FTIR spectrum measured in the near-IR range from 400 to 4000 cm^–1^ (Figure 5) with peak absorption bands at different bond stretches, shown as diverse peaks. Typically, in an IR spectrum, the 3550–3200 cm^–1^ bands relates to O–H stretching, H-bonded phenols, and alcohols; the 2950–2850 cm^–1^ peak corresponds to C–H (alkane) stretches; and 2830–2695 cm^–1^ is an indicative band for O=C–H (aldehydes) as one to two peaks with moderate intensity.^51,52^ Similarly, 2260–2100 cm^–1^ represents C≡C/C≡N as alkynyl and nitrile species. Also, the assignment of N–H band (as primary amines) links to 1652 cm^–1^, while 1609 cm^–1^ peaks show C=C medium–weak stretching vibrations of amines; the 1381–1025 cm^–1^ peak defines N–H (primary/secondary amine group) stretching; 1230 cm^–1^ band represents the characteristics of multiple C–O; and 1042, 1059, and 1089 cm^–1^ represent the C–N stretching of carboxylic acids, ethers/esters, and alcohols, respectively.^53,54^ Moreover, peaks between 775 and 658 cm^–1^ are assigned to C–OH, with usually broad C–O variable weak bending vibrations of alcohols and phenols.^54,55^

As depicted in Figure 5, our results for pure glycerol as well as silver NPs (with peak variations) highly correlate with the literature.^56−58^ For instance, absorption bands at 3100–3400 cm^–1^ indicate the O–H stretch of glycerol^52^ and those at 2695 cm^–1^ the aldehyde (C–H) group^45^ produced from glycerol.^59^ Expectedly, the peaks at 2933, 2879, and 2695 cm^–1^ of bare glycerol decreased significantly with the passage of silver NP formation, thus relating to the theory that glyceraldehyde (coming from glycerol)^45^ is involved in the reduction of silver metal to produce silver NPs, as predicted by Liu et al.^4^ Other than this, two main peaks at 3307 and 1638 cm^–1^ confirm the formation of silver NPs, with the latter peak indicating the characteristic C–O stretching. However, a similar peak appeared at 1641 cm^–1^ after the addition of PVP, which is allocated as an important characteristic absorbance peak of PVP for carbonyl (C=O) stretching.^60^ However, the band at 1049 cm^–1^ corresponds to several functional groups like alcohol, carboxylic acid, ester, ether, and anhydride,^57^ thus appearing in all sample spectra with varying intensities. Thus, these FTIR results for the formation of silver NPs are in agreement with those of Senthil et al., Ramli et al.,and Ahmed et al.^57,58,61^ Individual graphs of each component are also presented in Figure S4. Interestingly, FTIR analysis can not only give the qualitative (identification) analysis of materials but, with relevant standards, can also be used for quantitative (amount) analysis.^45^

### XRD Measurements

**Figure 6:**
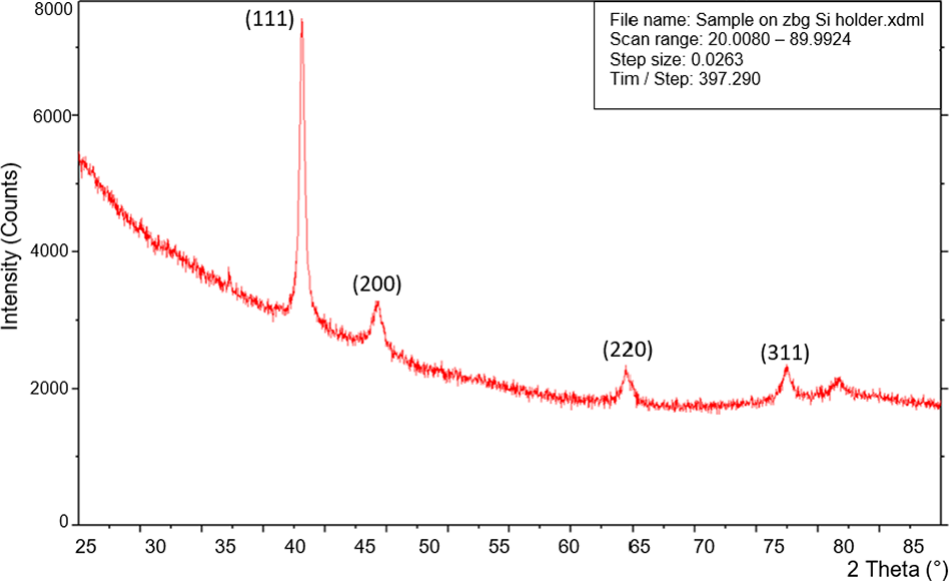
XRD patterns of the synthesized silver NPs in glycerol.

The XRD patterns were obtained to confirm the crystalline nature of the silver NPs. Figure 6 shows the diffraction peaks of the silver NPs which indicate the polycrystalline nature. The patterns disclose the peak diffraction corresponding to the fcc (face-centered cubic)-crystalline silver phase (JCPDS: 04-001-2617) with a characteristic split in the peaks exhibited at 2θ (i.e., 38.2°, 44.3°, and 64.7°, as 111, 200, and 220) planes, respectively. The data correlate with the previous findings of Mahitha et al.,^53^ Mehta et al.,^62^ dell’Erba et al.,^63^ and Senthil et al.^57^ The existence of these three peaks with a substantial intensity ascends from the silver NP location on the glass slide surface.

XRD is among the few popular techniques that can be used for both molecular as well as crystal structure analysis, qualitative/quantitative identification of chemical compounds, measurement of crystallinity, particle size, isomorphous substitutions, and so forth.^64^ Thus, XRD can evaluate the structural features of an extensive range of materials, and analysis of these materials essentially depends on the diffraction pattern formation from a molecule.^65^ For the identification of each material with a distinctive diffraction beam, a reference database library (Joint Committee on Powder Diffraction Standards––JCPDS) is used for the comparison of diffracted beams, and these patterns also describe the purity of the sample materials.^66,67^ In recent times, XRD has been applied for the characterization of numerous nanomaterials with certain properties.^68^ However, these diffracted patterns also explain if the material of the sample is pure or holds impurities.

Further, to determine the mean particle size through the XRD patterns, the calculation of peak position, peak intensity, and full width at half-maximum (FWHM) values was proceeded with 80% glycerol (random pick) using Scherrer’s formula, the equation for which is *d* = 0.9λ/βcosθ, where *d* is the mean diameter of the nanoparticles, λ is the wavelength of the X-ray radiation source, and β is the angular FWHM of the XRD peak at the diffraction angle θ.^69^ Using this equation, the estimated mean particle size from the major diffraction peaks was found to be 17 nm, correlating with our TEM and DLS data for the 80% glycerol sample of silver NPs. Therefore, the XRD study has confirmed that in our prepared sample, the resultant particles are of silver source.

### AFM Measurements

**Figure 7:**
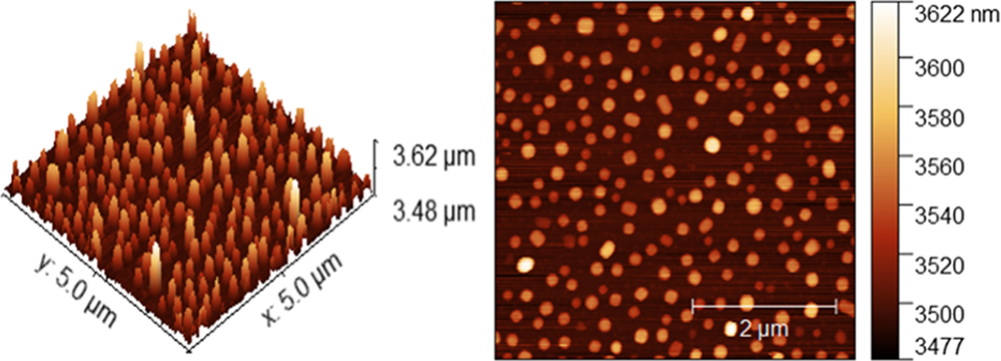
AFM analysis of silver NPs in 100% glycerol to investigate the dispersion and aggregation of nanomaterials, in addition to their size, shape, absorption, and structure.

For the validation of the particle structure, size, and aggregation status, AFM was used further to measure silver NPs at 100% glycerol concentration. Figure 7 illustrates the well-dispersed appearance of silver NPs, providing a high-density coverage on the silicon wafer substrate. Silver NPs appeared spherical in shape on AFM, which correlates with the observations from SEM images for the same sample percentage. However, SEM cannot offer any metrological information regarding the height of the nanoparticles, which was measured through AFM observations (Figure S5), along with the average length of the particles that appears in the similar range as observed in DLS (for the 100% sample).

For instance, an increase in hydrodynamic diameter (Z.avg), without affecting the polydispersity in DLS results, matches well with the slight increase in height of the silver NPs when detected by AFM cross-section analysis with no observation of aggregates. Thus, these measurements show complementarity and coherency in the results obtained from multiple techniques in our study, for the same sample. A study conducted by Ning et al. depicted the chemical synthesis of AgNPs as silver island films, using citrate and NaBH_4_ reduction of silver nitrate in water, and obtained silver nanoparticles of a vast size range, i.e., 8–500 nm.^70^ Interestingly, their AFM images of 100–200 nm give a good reference for our obtained results in the similar size range.

## Conclusions

In this work, a simple, quick, and reproducible method to acquire silver NPs with narrow size distribution, wide range of glycerol, and fine dispersion has been introduced using the green synthesis method, with glycerol as a reducing medium and PVP as a stabilizing agent, thus controlling the particle size as well as lowering the particle agglomeration. Further, this study offers a straightforward strategy to obtain nanoparticles with tunable size properties and formulated medium by glycerol at varying concentrations (10–100%) allows a remarkable progress in observing such desired size between 1.8 and 160 nm without prolong procedures. With the mentioned protocol, production of ultra-small-sized nanoparticles at large scale is potentially possible without additional time-consuming and costly steps/processes (such as the need of high temperature, pH adjustments, surfactants, or sonication).

## Supporting Information Available

Additional synthesis and characterization photographs of TEM analysis in correlation with DLS at different concentrations of glycerol (10–100%); DLS data for the glycerol percentages not observed under TEM due to similar size range, i.e., <10 nm; EDS-derived elemental map of silver NPs; FTIR absorption graphs before the beginning of silver NP synthesis; and additional AFM analyses (PDF)
