# Supplementary material for: Novel Size-Tunable and Straightforward Ultra-Small Nanoparticle Synthesis in a Varying Concentration Range of Glycerol as a Green Reducing Solvent
Source: ACS Omega. 2023 Jul 26;8(31):28456–66. doi: 10.1021/acsomega.3c02697 (PMC10413838; doi:10.1021/acsomega.3c02697)
Supplement: Supplementary file 1 — ao3c02697_si_001.pdf [file ao3c02697_si_001.pdf]

SUPPLIMENTARY FILE

A Novel Size-Tunable and Straightforward Ultra-Small Nanoparticles Synthesis in a Varying Concentration Range of Glycerol as a Green Reducing Solvent

Iqra Munir [a] and Gurkan Yesiloz[a,b*]

[a] *National Nanotechnology Research Center (UNAM)- Bilkent University, 06800, Cankaya-Ankara, Türkiye.*

[b] *Institute of Material Science and Nanotechnology, Bilkent University, 06800, Cankaya-Ankara, Türkiye.*

[*]E-mail: gurkan.yesiloz@bilkent.edu.tr

10% Gly **a** **b**

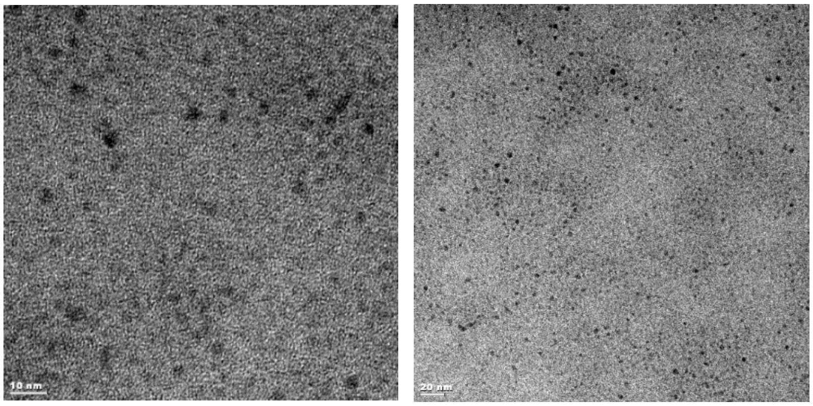

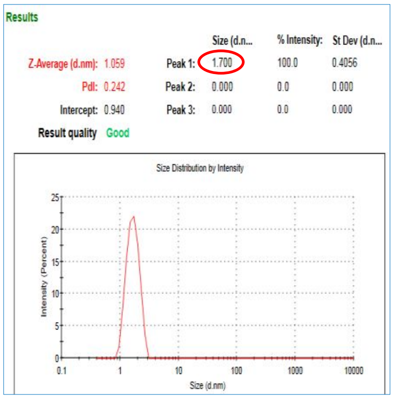

20% Gly **a** **b**

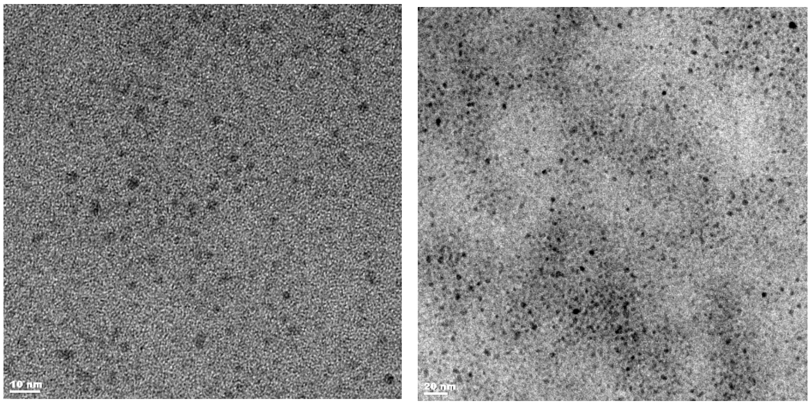

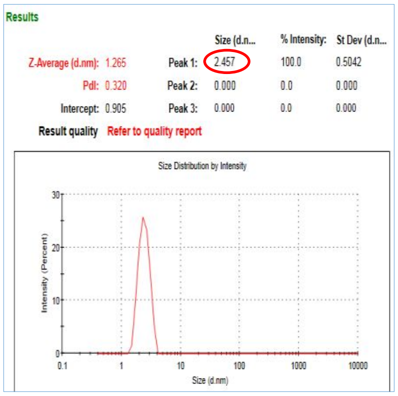

40% Gly **a** **b**

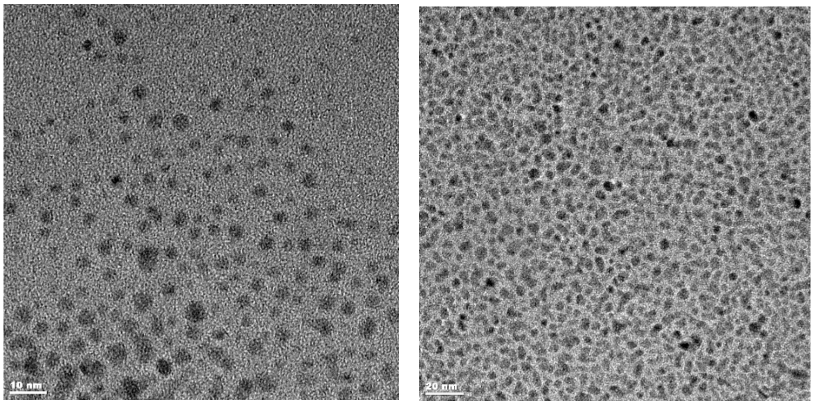

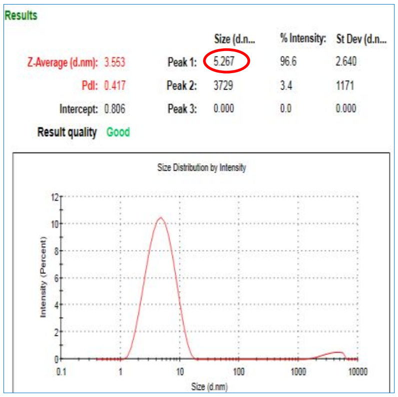

60% Gly a b

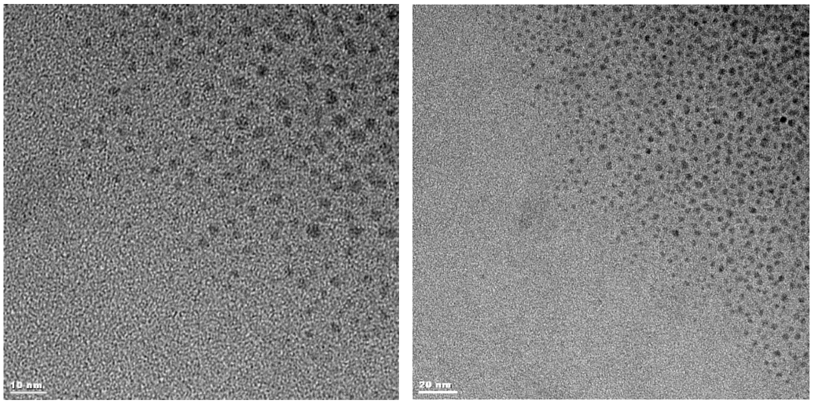

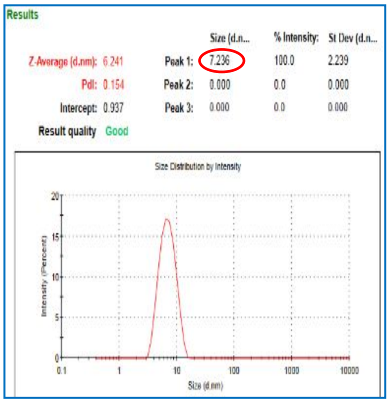

70% Gly a b

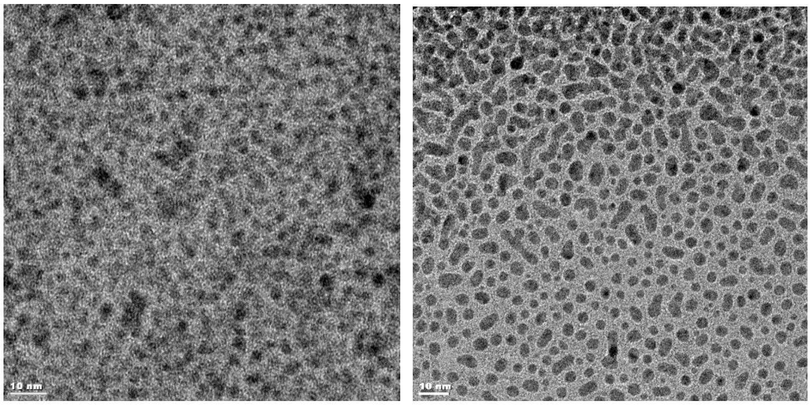

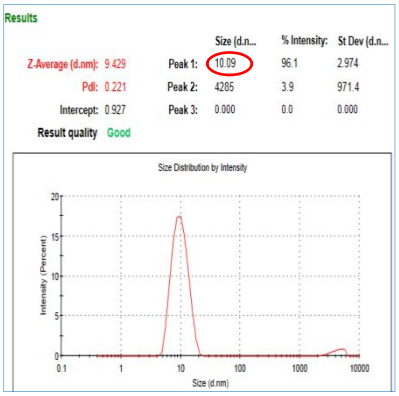

80% Gly a b

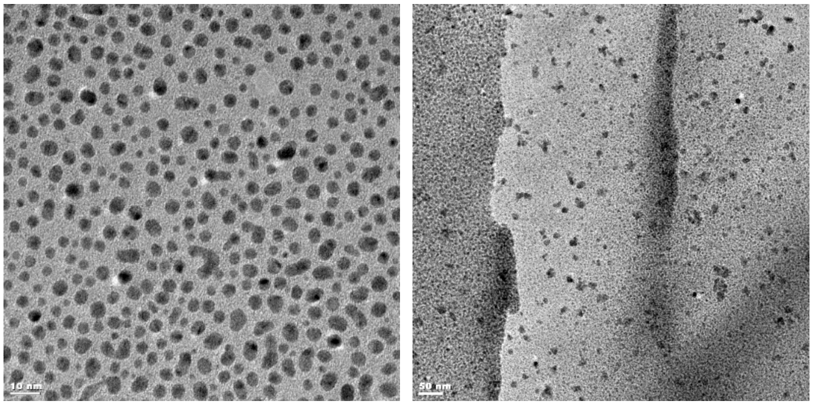

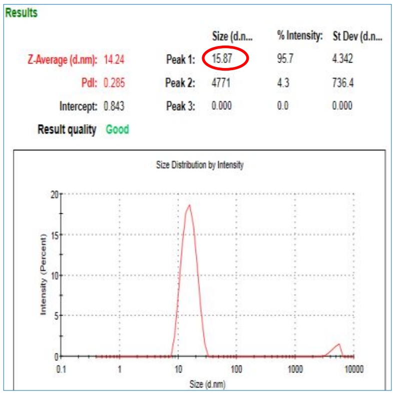

90% Gly **a** **b**

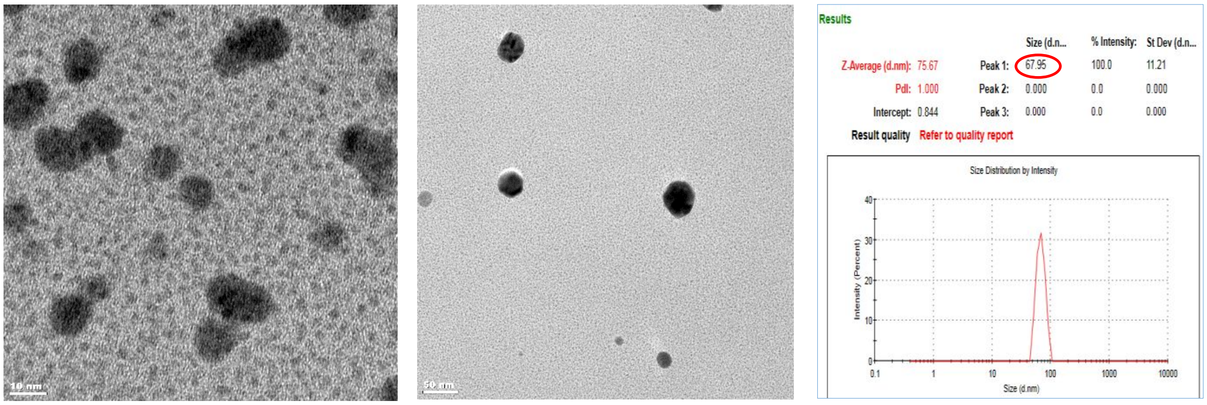

Figure. S1: TEM images of Silver NPs in glycerol (10-100%). (a) TEM images and (b) corresponding size measurements (by DLS) of synthesized silver NPs. Scale bars are 10nm-50nm.

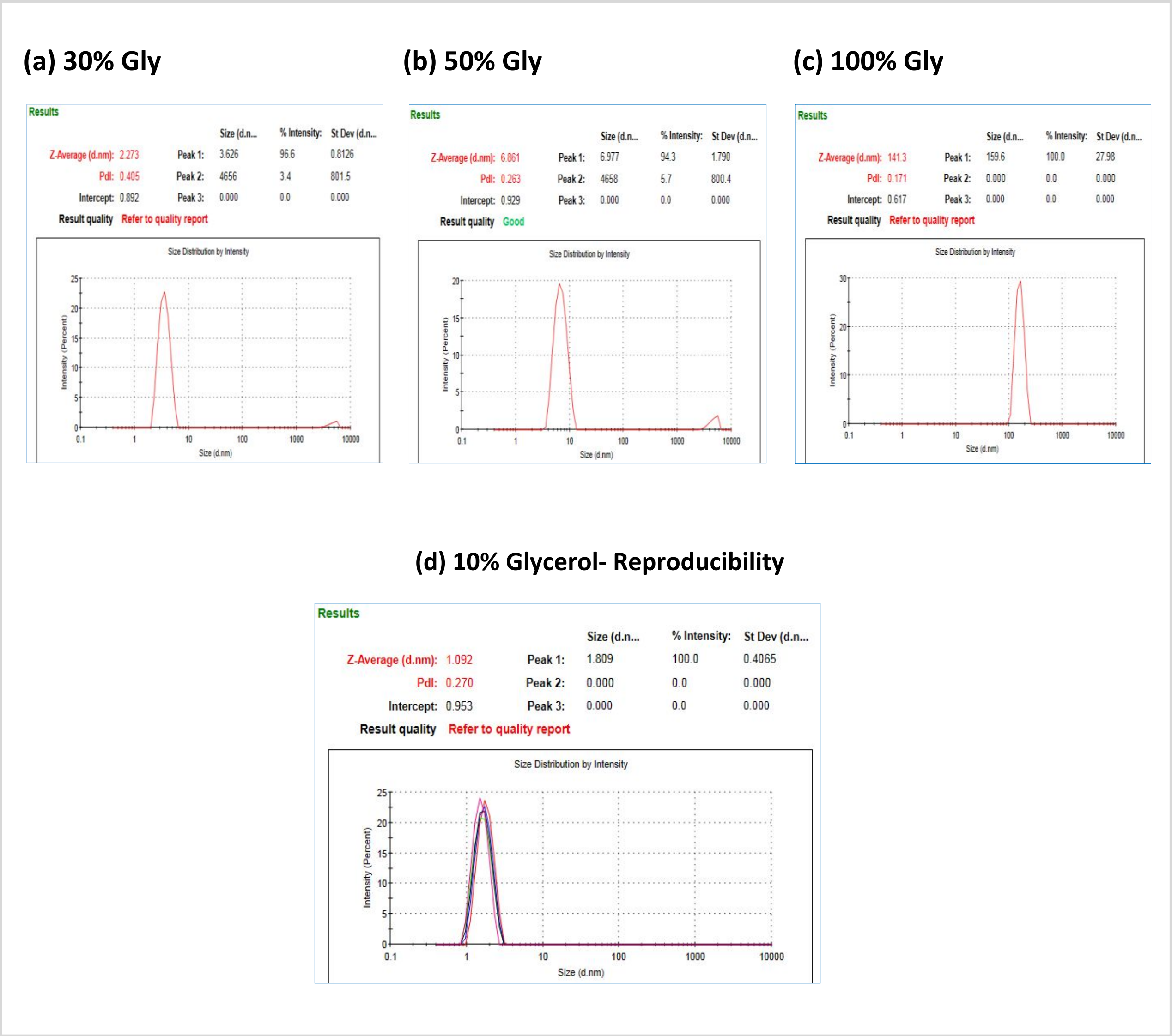

Figure. S2: (a-c) DLS data for the remaining percentages of glycerol (not observed under TEM due to similar size range i.e. <10nm). (d) Cumulative profile of 10% glycerol with 5 set of experiments, to check reproducibility.

(a)

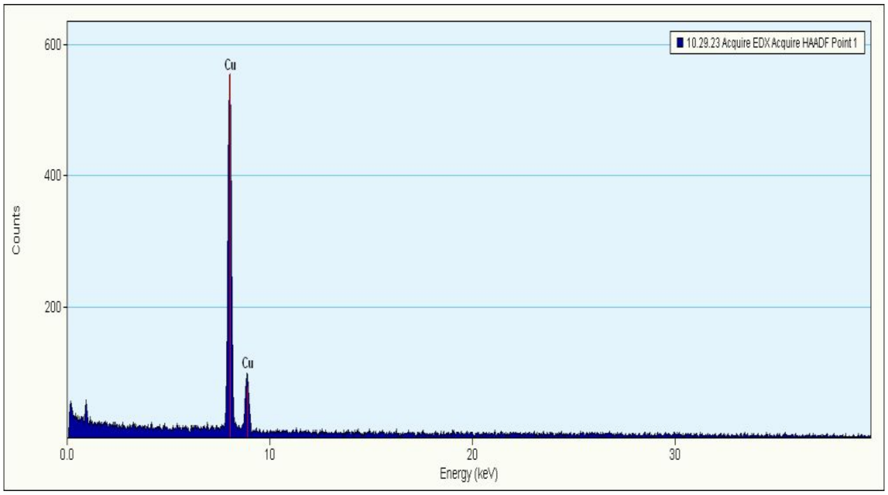

(b)

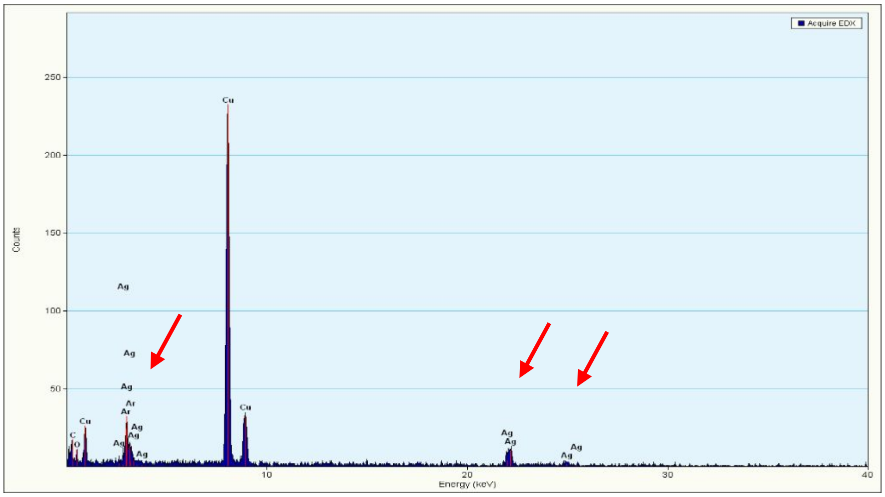

Figure. S3: EDS derived elemental map of Silver NPs sample (in glycerol) observed for TEM images (a) bare TEM Cu-grid, (b) TEM Cu-grid loaded with silver NPs (in 10% Glycerol).

(a)

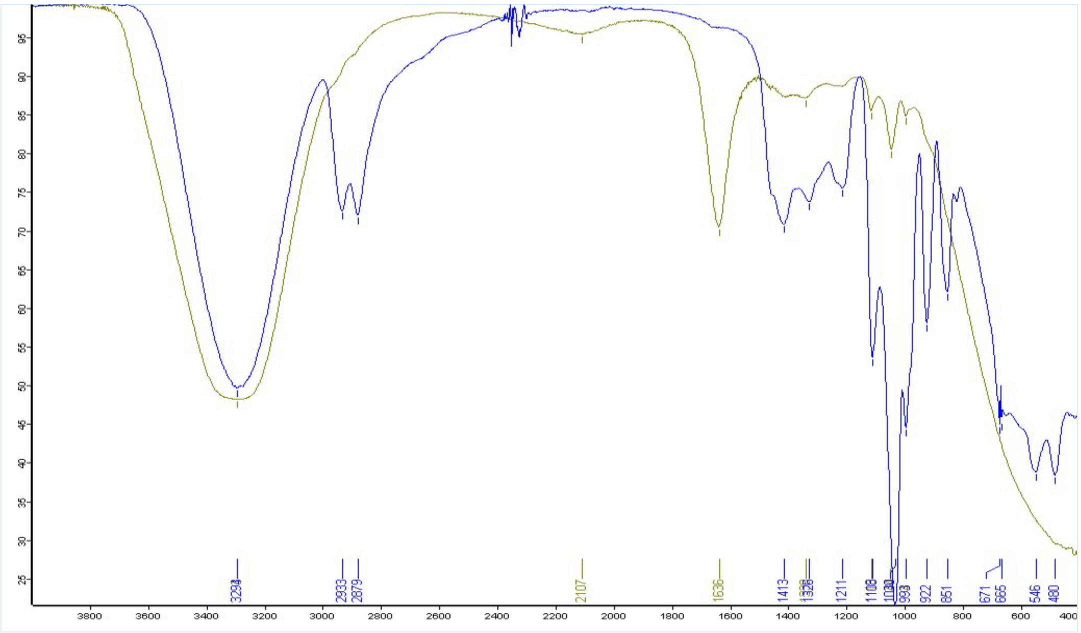

--- Gly-alone --- Gly + Silver Nitrate ($AgNO_3$)

(b)

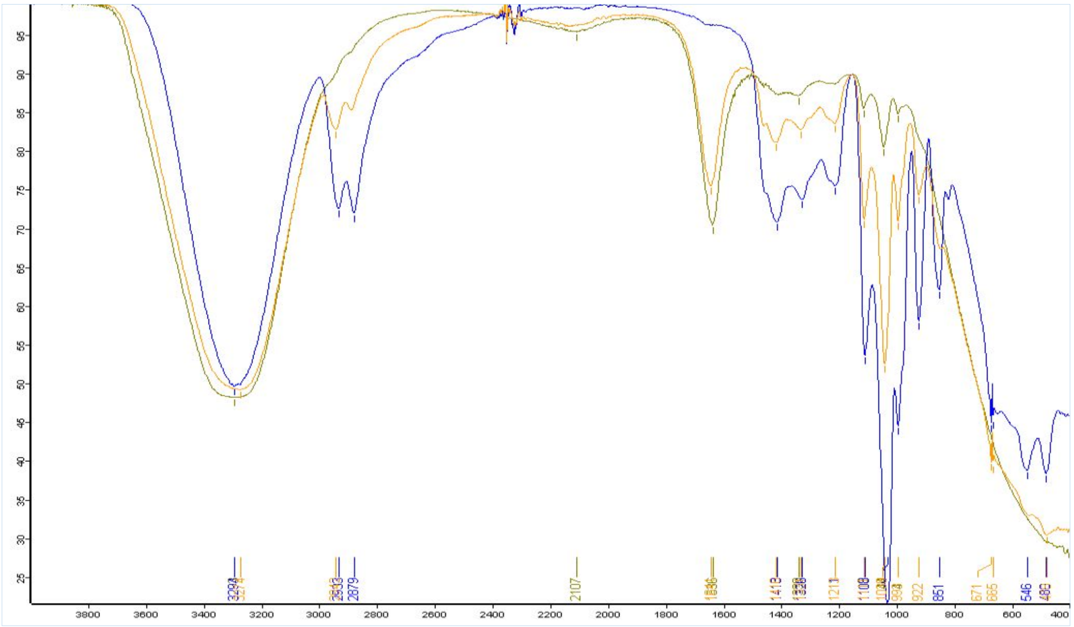

-- Gly-alone -- Gly + PVP -- Gly + Silver Nitrate ($AgNO_3$)

Figure. S4: FTIR Absorption spectrum: Individual graphs of synthesis material before the beginning of silver NPs synthesis. (a) Overlay spectrum of glycerol alone and glycerol silver nitrate (b) Overlay spectrum of glycerol alone, and after addition of PVP and $AgNO_3$.

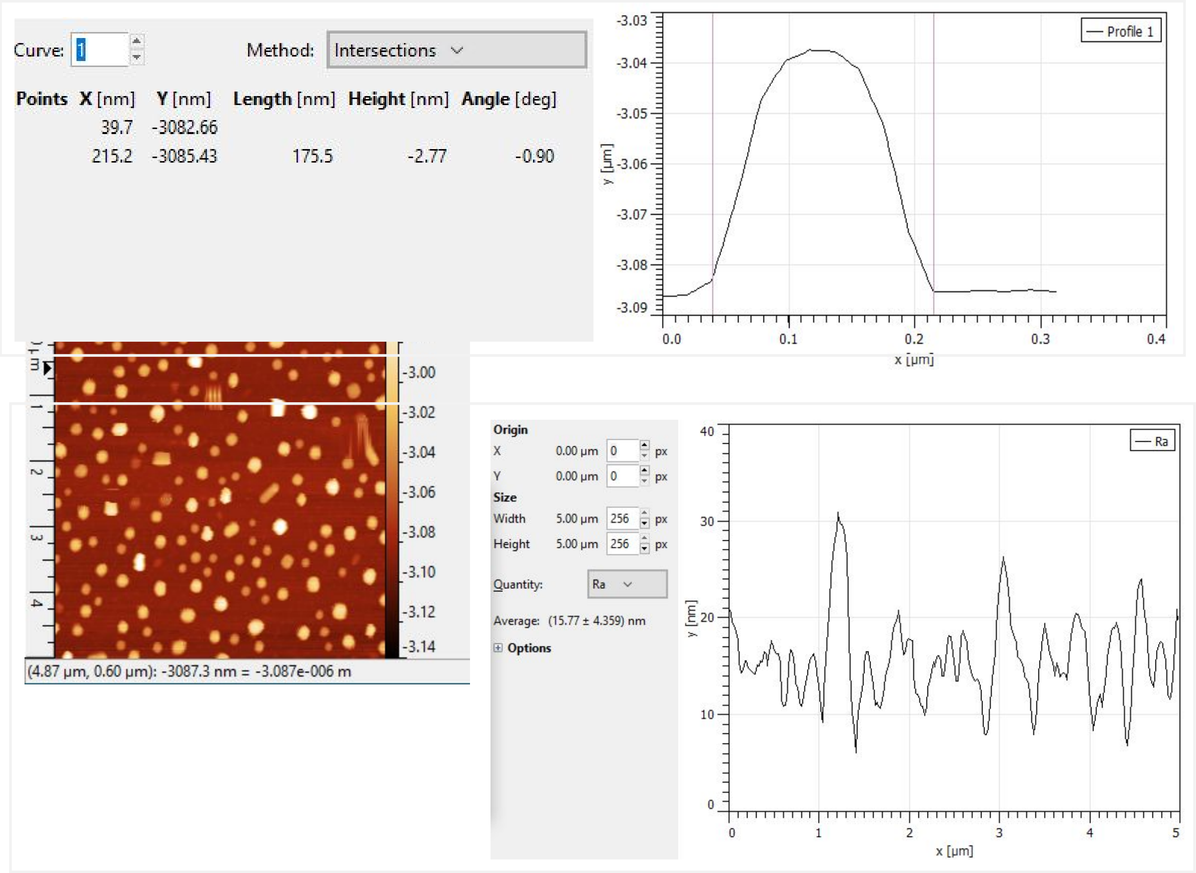

Figure. S5: AFM analysis of silver NPs in 100%Gly showing length and height measurements of the observed particles.
